# When Are Children Most Physically Active? An Analysis of Preschool Age Children’s Physical Activity Levels

**DOI:** 10.3390/children9071015

**Published:** 2022-07-08

**Authors:** Gema Díaz-Quesada, María de los Ángeles Gálvez-Calabria, Jonathan D. Connor, Gema Torres-Luque

**Affiliations:** 1 Department of Plastic, Music and Corporal Expression, Faculty of Humanities and Education Science, University of Jaén, 23071 Jaén, Spain; gmdiaz@ujaen.es (G.D.-Q.); magc0031@red.ujaen.es (M.d.l.Á.G.-C.); 2 Department of Sport and Exercise Science, James Cook University, Townsville, QLD 4811, Australia; jonathan.connor@jcu.edu.au

**Keywords:** physical activity, wearable technology, childhood

## Abstract

The levels of physical activity (PA) in the population have decreased, especially at an early age. The aims of the study were: to evaluate the percentage of children meeting PA recommendations for both genders, and to measure steps and PA level at different time intervals during the week. This was an observational cross-sectional study. Seventy-three schoolchildren (36 boys and 37 girls), aged two years (2.12 ± 0.46), were selected to participate in this study. Participants wore an “*Actigraph GT3X*” accelerometer for seven days to measure the minutes engaged in moderate-to-vigorous physical activity (MVPA) and step volume. The results show 100% of the children studied met the recommended 60 min/day of MVPA, and 50% achieved 120 min/day MVPA and 13,000 steps per day. No gender differences were found. The results of the analysis show a propensity for higher step volumes and PA values from Monday to Friday. In addition, subjects achieved higher step volumes and PA values during “School Time” than “Out-of-School Time”. Given that during “School Time” children showed higher PA and step values, schools represent an important place to help facilitate PA milestones. This study shows the need for further studies and interventions aimed at understanding and improving children’s PA levels at an early age.

## 1. Introduction

The regular practice of physical activity (PA) is associated with multiple health benefits in children at different levels (social, psychological and physical), as early PA is essential for healthy development and growth in childhood [1,2]. PA is favorably related to the development of motor skills, mental health, cardiometabolic health, cognitive skills and osseous and skeletal health [3,4]. Children around 5 years of age who are inactive tend to continue their physical inactivity during childhood and adolescence [5]. In turn, PA is essential to fight childhood obesity, which is one of the most severe public health problems of the 21st century [5]. Currently, almost 40% of Spanish children are obese or overweight [6]. Based on the latest World Health Organization (WHO) report, the incidence of obesity in this group has grown alarmingly over the last decade in Spain, which has the highest rate of childhood obesity in Europe [7].

Various organizations have established minimum recommendations of 180 min of PA per day at any intensity (light PA (LPA), moderate-to-vigorous PA (MVPA) or vigorous PA (VPA)), particularly during the first stage of preschool education [8,9]. In the second cycle of preschool education, of these 180 min of PA, recommendations propose that at least 60 min should be spent engaging in MVPA [10,11], increasing to 120 min of daily MVPA for ages 2–3 years and older [12]. An additional recommendation for measuring PA is recording the volume of steps achieved, with recommendations of between 10,000 and 14,000 steps for this age group [13,14]. However, according to previous studies, some preschool populations did not reach these health recommendations regarding the level of PA [15,16]. For example, data from Japanese children aged 3–4 years show they only reached around 34–35% of these recommendations [17]. Other studies examined the differences in levels of PA that exist in terms of gender, highlighting that boys are often significantly more active than girls in all categories of PA intensity [18,19]. Another study found that girls spent 19% fewer minutes in MVPA and 5% fewer minutes in LPA than boys during preschool hours [20]. Likewise, girls only spent 43.0 min/day in MVPA, while boys spent 56.8 min/day in MVPA [21].

However, a notable limitation to some research in this area has been the lack of consideration for various times throughout the day, different situations and spaces, which may facilitate PA amongst children. For example, in the school context, some studies have shown that preschool children who attend school obtain the same amount of MVPA as those who are not in school [22]. In contrast, more recent research showed Spanish schools have a modest contribution of 24% to the recommended minimum PA levels [19]. These recent studies highlight that children’s school settings are valuable environments for helping to increase the level of PA [23,24]. In particular, the outdoor environment and the playground can provide a comprehensive role in the promotion of PA and the decrease in sedentary behavior in this age group [2,25]. Despite the modest contribution by the school environment in achieving recommended levels of PA, different studies have also shown that preschool children perform more MVPA on weekdays compared to weekend days [26,27]. Another study reflects that Chinese preschool-aged children spent significantly more time engaging in vigorous activities on weekdays than on weekends [28]. However, there has been less empirical focus on Spanish children in this context.

Most studies examining the level of PA intensity are based on primary and secondary educational stages, whereas studies at younger ages remain scarce despite the significant health benefits of PA in younger children [16]. Furthermore, few studies have examined the time periods throughout the week which may promote or impede children engaging in physical activity. Therefore, a novel aspect of this current study is the target population of infant children, given that this age group has not been studied due to the difficulties of gaining access at an early age. This study suggests that depending on gender or the moment in the day, children achieved different levels of PA and number of steps. Therefore, the present study aimed to evaluate the compliance with PA recommendations based on gender and to determine the PA levels throughout the different time intervals of the day in children in the first cycle of preschool education.

## 2. Materials and Methods

### 2.1. Design

A cross-sectional observational study was carried out to compile data on children’s PA at several times of the day. Data were collected at a predetermined time over seven consecutive days during a normal school week within the same population group.

### 2.2. Participants

A total of 73 children in kindergarten (age 2.12 ± 0.46; gender = 36 boys (49.31%) and 37 girls (50.69%); and BMI = 17.06 ± 4.22) were recruited for this study. None of the participants had a physical disability or psychiatric illness that prevented them from participating in the study. The children came from two day-care centers located in Jaén (Spain). The aims of the study were explained to both the children and their families, and parents signed an informed consent form for their child to participate. The Ethics Committee of the local institution (University of Jaén, Spain (JUN.17/6)) approved this work.

### 2.3. Procedure

The participants were randomly recruited. Only data from those subjects who were wearing the activity tracker daily were used. Participants wore the Actigraph GT3X accelerometer (Actigraph, Pensacola, FL, USA), during seven continuous days of a routine school week [29,30]; the device collects and stores information on steps and PA time in 3 orthogonal axes: vertical (Y), horizontal right–left (X) and horizontal front–back (Z). It also contains the “magnitude vector”, which is the square root of the sum of each axis squared, which makes the device valid for measuring PA [31]. The GT3X device was placed on the right hip, midway over the iliac crest, using an adjustable band [32,33]. In addition to verbal instructions during device placement, families were provided with an explanatory sheet on the placement and use of the device, indicating its removal during water activities (swimming or showering) and for sleeping. The data were stored with an interval of 15 s, an interval recommended for quantifying PA in schoolchildren [34]. PA levels were obtained by classifying the counts recorded according to the cut-off points of [35]: sedentary activity level < 200 counts/15 s; LPA ≥ 200–419 counts/15 s; MPA ≥ 420 counts/15 s; and VPA ≥ 842 counts/15 s, as previously used in other articles such as Pate et al., 2016 [36]. Sixty seconds of continuous zero activity was considered invalid, and a recording time of ≥10 h/day and ≥5 days during the week (four weekdays and one weekend day) was considered valid [37]. The information was expressed in minutes per day (min/day) by percentage (%). All data were subsequently analyzed with Actilife 6.0 Software, Scientific Advisory Board, Florida, United States [38], which analyzes in a general and partial way the data requested according to the PA levels of Pate et al., [36]. PA time was recorded and categorized based on the school days (Monday to Friday—Midweek; Saturday to Sunday—Weekend) and times (School Time—8:00 to 14:00 h; Out-of-School Time (14:01 to 23:00 h).

In order to quantify MVPA, the data were measured as minutes per day (min/day), while steps were measured as steps per day (steps/day). To investigate how many participants were achieving the appropriate amount of physical activity, cut-offs were set at 60 min of MVPA [39], 120 min of MVPA and 13,000 steps, based on previous age reference values for children of a similar age in the study sample [13,14].

### 2.4. Statistical Analysis

Descriptive analysis was conducted and is presented in Table 1 as means and standard deviation. Prior to analysis, a Kolmogorov–Smirnov normality test was performed to verify normal distribution of data. Thereafter, a series of inferential statistical tests were conducted to compare groups. For independent samples, *t*-test was then calculated to compare gender (boys vs. girls). When comparing differences between different moments of the week and times of the day (Midweek vs. Weekend and School Time vs. Out-of-School Time), a Student’s paired samples *t*-test was adopted. Finally, Bayesian inferences were conducted on both *t*-tests for paired samples and independent samples. The level of statistical significance was set at *p* ≤ 0.05. Analyses were conducted with the spreadsheet called Jamovi 2.2.2. and the IBM SPSS Statistics 25.0 software for Windows.

## 3. Results

Table 1 shows the descriptive characteristics of the participants. Table 2 shows the Bayes factor used for comparison of PA recommendations by gender. Table 3 shows the Student’s paired samples *t*-test and Bayesian methodology used to compare differences for PA levels, and what percentage of the PA recommendations participants were achieving between “Monday to Friday” and the “Weekend”. Table 4 shows the Student’s paired samples *t*-test (Bayesian and frequentist inferences), used to compare differences for PA levels and percentage of the PA recommendations participants were achieving between “School Time” and “Out-of-School Time”.

When analyzing the data descriptively, Table 2 indicates that throughout the “week” (Monday to Friday and the Weekend), participants were found to meet around 100% of the 60 min/day recommendations, while only around 50% achieved the recommended 120 min/day and 13,000 steps/day. When analyzing the “School Time”, the recommended 60 min/day was achieved by around 50%, while the recommended 120 min/day was achieved by around 40% and the recommended 13,000 steps/day was achieved by around 25%. Regarding “Out-of-School Time”, approximately 20% achieved the recommended 60 min/day; those achieving the recommended 120 min/day was approximately 10%, and those achieving the recommendations of 13,000 steps/day was around 1%.

Statistically significant differences could be seen on the weekdays (Monday to Friday) for MVPA min/day and in Accomplishment Recommendations (AR) were found Monday to Friday for the 60 MVPA min/day recommendation (%). Boys had higher mean values than girls for the AR Monday to Friday of 120 MVPA min/day (%) (*p* ≤ 0.005). The Student’s paired samples *t*-test and Bayesian methodology in Table 3 show a propensity for higher results in “Monday to Friday”, compared to the “Weekend”, although no robust evidence in favor of H_1_ (differences between “Monday to Friday” and “Weekend”) and no statistically significant differences were found. Nevertheless, approximately 50% of the recommendations of steps and MVPA of 120 min/day were met.

Data analysis using the Bayesian statistic revealed anecdotal evidence (BF10 1–3) in favor of H_1_ in the following variables: Monday to Friday MVPA min/day, Accomplishment Recommendations (AR) Monday to Friday 60 MVPA min/day (%) and AR Monday to Friday 120 MVPA min/day (%) compared between “Boys” and Girls” in Table 2 (Boys > Girls). Statistically significant differences in Monday to Friday MVPA time and Monday to Friday recommendations of 60 and 120 min MVPA per day (*p* < 0.05) were shown in the frequentist analysis. Also, Bayesian analysis showed extreme evidence (BF10 > 100) in favor of H_1_ in the following variables: steps/day, MVPA min/day, AR 60 MVPA min/day (%), AR 120 MVPA min/day (%) and AR 13,000 steps/day (%) when compared between “School Time” and “Out-of-School Time” in Table 4 (School Time > Out-of-School Time). Statistically significant differences in steps/day and MVPA time, recommendations of 60 and 120 min MVPA per day and recommendations of 13,000 steps/day (*p* < 0.001) were shown in the frequentist analysis.

## 4. Discussion

The present study assessed children’s PA levels by gender in the first cycle of preschool education, throughout the different periods of the day and week, and examined whether they meet current recommendations. The main results of this study show that the current population met the recommendations of 60 min/day of MVPA and only 50% met the recommendations of 120 min/day of MVPA and 13,000 steps/day.

While some literature reviews have shown that preschool children do not met PA recommendations [15,17], the results of this study show 100% compliance in 60 min/day of MVPA. This finding is also in line with other previous empirical studies showing low physical activity levels [40,41,42]. There were, however, some toddlers who met the recommendations of 3 h of PA at any intensity [40,41]. Additionally, these results show only 50% compliance of 120 min/day was achieved, which is surprisingly given that some other studies have shown a 20–30% compliance rate [43,44,45]. In terms of steps, the results show only 50% compliance with 13,000 steps/day, whilst other investigations have reported lower compliance values with steps/day [4,46]. Therefore, the different results accessed in this study indicate that, at these ages, the daily PA recommendations are not being met. This is a problem, because in the early ages, where they are very active and are picking up healthy lifestyle habits, they do not move enough, which can lead to health problems throughout childhood.

This study shows that boys had a higher tendency to engage in MVPA than girls; however, there were no gender differences in PA behavior other than the MVPA values, which were higher in boys than girls on weekdays. It is important to note that gender differences in PA behavior are still controversial; on the one hand, some studies indicate that boys have higher MVPA than girls [4,44], while others indicate that there are no differences [22]. This absence of differences may be due to the age of the sample studied, as in the present study, the children were two years old, a stage close to the development of bipedal vertical locomotion and with greater homogeneity at the motor level between genders [47]. In turn, in analyzing other studies, it seems that more significant differences between genders are found when a greater number of subjects are studied or when the duration of the research is longer [17,18]. So, it would be interesting to pursue further studies at these ages to see what happens with different samples.

On the other hand, in the comparison of MVPA data from Monday to Friday versus MVPA from Saturday to Sunday (Table 3), it was shown that there was no difference in the level of MVPA between these periods of the week. However, there are other studies with significant results showing levels of MVPA were higher on weekdays than on weekends [26,27,28]. These inconsistencies could be due to different factors, including cultural and/or geographic differences between continents, sociodemographic and temporal characteristics, parental education level or socio-economic status [48,49]. Future studies should look to further explore these factors as potential moderators of PA in young children.

The present study has investigated the comparison between MVPA performed in School Time versus MVPA performed in Out-of-School Time. In this case, the results show significant differences; the sample students performed higher levels of MVPA in School Time than in Out-of-School Time (Table 4). Although some PA recommendations were not met, it is important to highlight the fact that the results of this study show more MVPA in School Time, leading to the conclusion that schools are facilitating PA to some extent. Therefore, it is vital to continue emphasizing the importance of the school as a place to promote PA. In Spain, the Organic Law 2/2006 on education, which came into force on 3 May [50], states that early childhood education is an educational stage that spans from birth to 6 years of age, and it is divided into two cycles, with the first cycle including birth to 3 years of age, and the second cycle spanning from 3 years of age to 6 years of age. Due to the approval of this law, early childhood education became the first educational stage of the Spanish system. However, other research in preschool [51,52] and older children [53] in Spain [54] showed that children engage in more MVPA during Out-of-School Time than during School Time. It is suggested that the school setting offers an ideal environment to provide PA opportunities and intervene to increase daily PA and meet daily recommendations. Practical recommendations for educators and practitioners should consider that, when building or renovating facilities, providing spaces that promote PA is paramount for children to meet the recommended PA levels [55,56].

The main limitation of this study was the reduced sample size due to the limited accessibility at early ages. Additionally, caution is required when generalizing these results to other population groups.

## 5. Conclusions

These findings can provide useful input for the implementation of educational programs and strategies to increase the levels of PA in the schools. Future research is required to analyze PA in children because of the importance of early intervention, when good health habits related to PA and sport can be facilitated.

## 6. Practical Applications

It is necessary to search for solutions to improve the results of PA in the schools, such as those studies focusing on improving intrinsic motivation towards PA [57,58]. It has been proven that intrinsic motivation is associated with a longer time spent performing MVPA, and the teachers who teach physical education classes play a fundamental role here, since they are in charge of motivating the students [59,60].

These studies should serve as a guide for early childhood education teachers, to emphasize the influence that they have on students and everything they have at their fingertips to teach and motivate students throughout their growth and development. An example of PA intervention is PASE (Outdoors, Health and Environment), a program carried out in Montreal [61], whose purpose is for students to go outside the school to study their subjects, supplemented with different outdoor learning activities (writing a poem, observing flora or introduction to canoeing).

Brain breaks, a web-based PA break that fosters students’ health and learning, is another intervention designed particularly for the classroom context [62]. It was introduced by HOPSports and is applied to motivate participants to improve their theory lessons and to offer them the possibility of being physically active during breaks, while learning new skills such as music, language and art or exploring different cultures [63].

There should also be efforts to increase awareness of parents regarding the importance of young preschool-aged children being physically active outside of school hours.

## Figures and Tables

**Table 1:** Characteristics of participants.

	Total (*n* = 73)
Age (yr), mean (SD)	2.12 ± 0.46
Gender, n males (%)	36 (49.31)
Weight (kg)	14.61 ± 1.85
Height (m)	0.92 ± 0.08
BMI (kg/m^2^)	17.06 ± 4.22

BMI: Body Mass Index Corporal; kg: kilograms; m: meters; SD: standard deviation; yr: years.

**Table 2:** Descriptive characteristics and physical activity recommendations by gender.

Variable	Total (*n* = 73)	Boys (*n* = 36)	Girls (*n* = 37)	*p*	Error %	Bayes Factor	δ
Age (yr), mean (SD)	2.12 ± 0.46	2.10 ± 0.40	2.14 ± 0.52	0.727	0.01	BF_01_ = 3.568	−0.074
Weight (kg)	14.61 ± 1.85	14.47 ± 1.92	14.76 ± 1.80	0.573	0.01	BF_01_ = 3.213	−0.125
Height (m)	0.92 ± 0.08	0.93 ± 0.07	0.92 ± 0.09	0.654	0.01	BF_01_ = 3.378	0.097
BMI (kg/m^2^)	17.06 ± 4.22	16.85 ± 3.01	17.27 ± 5.21	0.718	0.01	BF_01_ = 3.454	−0.076
Week steps/day	8868.56 ± 3205.12	9467.37 ± 3430.09	8285.95 ± 2898.19	0.116	0.00	BF_01_ = 1.404	0.322
Week MVPA (min/day)	63.01 ± 24.00	67.39 ± 26.64	58.75 ± 20.61	0.125	0.00	BF_01_ = 1.480	0.315
AR Week 60 min/day (%)	105.02 ± 40.00	112.31 ± 44.39	97.92 ± 34.35	0.125	0.00	BF_01_ = 1.480	0.316
AR Week 120 min/day (%)	52.51 ± 20.00	56.15 ± 22.20	48.96 ± 17.17	0.125	0.00	BF_01_ = 1.480	0.311
AR Week 13,000 steps/day (%)	68.22 ± 24.65	72.83 ± 26.39	63.74 ± 22.29	0.116	0.00	BF_01_ = 1.404	0.317
Monday to Friday steps/day	6404.24 ± 2153.86	6762.03 ± 2221.07	6056.11 ± 2056.58	0.163	0.00	BF_01_ = 1.768	0.281
Monday to Friday MVPA (min/day)	65.10 ± 22.02	70.08 ± 23.15	60.27 ± 19.99	0.056	0.00	BF_10_ = 1.194	0.392
AR Monday to Friday 60 min/day (%)	108.51 ± 36.70	116.79 ± 38.58	100.45 ± 33.32	0.056	0.00	BF_10_ = 1.194	0.396
AR Monday to Friday 120 min/day (%)	54.25 ± 18.35	58.40 ± 19.29	50.22 ± 16.66	0.056	0.00	BF_10_ = 1.194	0.397
AR Monday to Friday 13000 steps/day (%)	49.26 ± 16.57	52.02 ± 17.09	46.59 ± 15.82	0.163	0.00	BF_01_ = 1.768	0.281
Weekend steps/day	6160.82 ± 3276.43	6763.33 ± 3509.66	5574.59 ± 2962.79	0.122	0.00	BF_01_ = 1.453	0.315
Weekend MVPA (min/day)	61.23 ± 31.42	67.35 ± 32.45	55.26 ± 29.62	0.101	0.00	BF_01_ = 1.271	0.334
AR Weekend 60 min/day (%)	102.04 ± 52.37	112.26 ± 54.08	92.11 ± 49.37	0.101	0.00	BF_01_ = 1.271	0.330
AR Weekend 120 min/day (%)	51.02 ± 26.19	56.13 ± 27.04	46.05 ± 24.68	0.101	0.00	BF_01_ = 1.271	0.340
AR Weekend 13,000 steps/day (%)	47.39 ± 25.20	52.03 ± 27.00	42.88 ± 22.79	0.122	0.00	BF_01_ = 1.453	0.318
School Time steps/day	3075.50 ± 1059.38	3281.06 ± 1015.54	2875.51 ± 1076.36	0.102	0.00	BF_01_ = 1.286	0.336
School Time MVPA (min/day)	29.98 ± 11.62	31.83 ± 12.76	28.18 ± 10.25	0.182	0.00	BF_01_ = 1.898	0.276
AR School Time 60 min/day (%)	49.97 ± 19.37	53.05 ± 21.27	46.97 ± 17.08	0.182	0.00	BF_01_ = 1.898	0.270
AR School Time 120 min/day (%)	41.64 ± 16.14	44.21 ± 17.72	39.14 ± 14.23	0.182	0.00	BF_01_ = 1.898	0.273
AR School Time 13,000 steps/day (%)	23.66 ± 8.15	25.24 ± 7.81	22.12 ± 8.28	0.102	0.00	BF_01_ = 1.286	0.333
Out-of-School Time steps/day	1119.90 ± 733.59	1235.18 ± 836.45	1007.74 ± 608.17	0.187	0.01	BF_01_ = 1.937	0.266
Out-of-School Time MVPA (min/day)	11.06 ± 7.24	12.08 ± 8.24	10.06 ± 6.06	0.234	0.01	BF_01_ = 2.232	0.238
AR Out-of-School Time 60 min/day (%)	18.43 ± 12.07	20.14 ± 13.74	16.76 ± 10.09	0.234	0.01	BF_01_ = 2.232	0.241
AR Out-of-School Time 120 min/day (%)	9.21 ± 6.03	10.07 ± 6.87	8.38 ± 5.05	0.234	0.01	BF_01_ = 2.232	0.243
AR Out-of-School Time 13,000 steps/day (%)	0.09 ± 0.06	0.09 ± 0.06	0.08 ± 0.05	0.234	0.01	BF_01_ = 2.232	0.243

AR: Accomplishment Recommendations; BMI: Body Mass Index Corporal; min: minutes; MVPA: moderate-to-vigorous physical activity.

**Table 3:** PA differences in kindergarten children between “Monday to Friday” and “Weekend”.

Variable	Monday to Friday	Weekend	*p*	Error %	Bayes Factor	δ
Steps/day	6404.24 ± 2153.86	6160.82 ± 3276.43	0.382	1.49 × 10^−5^	BF_01_ = 5.357	0.099
MVPA (min/day)	65.10 ± 22.02	61.23 ± 31.42	0.151	1.05 × 10^−5^	BF_01_ = 2.859	0.159
Recommendations 60 min/day (%)	108.51 ± 36.70	102.04 ± 52.37	0.151	1.05 × 10^−5^	BF_01_ = 2.859	0.159
Recommendations 120 min/day (%)	54.25 ± 18.35	51.02 ± 26.19	0.151	1.05 × 10^−5^	BF_01_ = 2.859	0.162
Recommendations 13,000 steps/day (%)	49.26 ± 16.57	47.39 ± 25.20	0.382	1.49 × 10^−5^	BF_01_ = 5.357	0.098

min: minutes; MVPA: moderate-to-vigorous physical activity.

**Table 4:** PA differences in kindergarten children between “School Time” and “Out-of-School Time”.

Variable	School Time	Out-of-School Time	*p*	Error %	Bayes Factor	δ
Steps/day	3075.50 ± 1059.38	1119.90 ± 733.59	<0.001	6.35 × 10^−28^	BF_10_ = 6.12 × 10^22^	1.882
MVPA (min/day)	29.98 ± 11.62	11.06 ± 7.24	<0.001	3.37 × 10^−27^	BF_10_ = 5.168 × 10^21^	1.802
Recommendations 60 min/day (%)	49.97 ± 19.37	18.43 ± 12.07	<0.001	3.37 × 10^−5^	BF_10_ = 5.168 × 10^21^	1.799
Recommendations 120 min/day (%)	41.64 ± 16.14	9.21 ± 6.03	<0.001	5.79 × 10^−5^	BF_10_ = 1.211 × 10^27^	2.251
Recommendations 13,000 steps/day (%)	23.66 ± 8.15	0.09 ± 0.06	<0.001	3.04 × 10^−37^	BF_10_ = 4.089 × 10^33^	2.883

min: minutes; MVPA: moderate-to-vigorous physical activity.

## Data Availability

Not applicable.
